# Integrative genomic and transcriptomic insights into the biocontrol activity of *Pseudomonas* sp. PF05 against *Fusarium oxysporum*

**DOI:** 10.3389/fmicb.2026.1784504

**Published:** 2026-04-10

**Authors:** Francesca Di Rico, Gabriele Bellotti, Francesco Vuolo, Edoardo Puglisi

**Affiliations:** 1 Department for Sustainable Food Process, Università Cattolica del Sacro Cuore, Piacenza, PC, Italy; 2 Sacco System, Cadorago, CO, Italy

**Keywords:** biocontrol, biological disease control, functional gene expression, functional genomics, microbe-microbe interactions, phytopathogen, pseudomonads

## Abstract

Microbial biological control agents (MBCAs) represent a sustainable alternative to chemical pesticides for the management of soil-borne fungal pathogens. Among them, fluorescent pseudomonads are widely recognized for their biocontrol potential, yet the precise mechanism of action and the molecular determinants underlying strain-specific antifungal activity remain incompletely understood. In this study, we investigated the genetic and transcriptional bases of biocontrol efficacy in *Pseudomonas* sp. PF05, a strain exhibiting strong antagonism against the phytopathogen *Fusarium oxysporum*, by integrating comparative genomics and transcriptomic analyses with those of a closely related but weakly antagonistic strain *Pseudomonas frederiksbergensis* PF4.89. Genome analyses revealed that strain PF05 harbors an expanded repertoire of genes associated with secondary metabolite biosynthesis, including phenazine metabolism, polyketide biosynthesis, and non-ribosomal peptide-related functions, compared with PF4.89. Transcriptomic profiling revealed that, during direct interaction with *F. oxysporum*, PF05 did not primarily activate canonical antifungal biosynthetic pathways, but instead mounted a coordinated adaptive response centered on efflux systems, metabolic reprogramming, detoxification processes, and regulatory networks. This response was accompanied by repression of respiratory activity, iron uptake, energy-intensive transport and secretion systems, indicating a strategic reprogramming aimed at stress tolerance and metabolic efficiency. In contrast, PF4.89 displayed a basal transcriptional profile characterized by a more limited regulatory engagement and reduced basal activation of pathways associated with microbial competition. Together, these results indicate that effective biocontrol in *Pseudomonas* sp. PF05 arises from the interplay between genomic potential and dynamic transcriptional regulation, rather than from the expression of individual antifungal genes alone. This work provides new insights into the multifaceted strategies underpinning microbial biocontrol and highlights regulatory and metabolic flexibility as key traits for the development of reliable MBCAs.

## 1 Introduction

Plant pathogens pose one of the most serious threats to agricultural production. At global scale, pathogens and pests are responsible for substantial yield losses, which for major food crops are estimated to range between 17 and 40%, depending on region and assessment method. Beyond yield reduction, plant pathogens also compromise product quality and impose a considerable economic burden, with direct and indirect losses estimated to reach tens of billions of dollars annually (Möhring et al., 2025; Pandit et al., 2022). Climate change and global trades are increasing the incidence and geographic spread of phytopathogens, increasing the risk of disease introduction into regions previously unaffected. This is particularly relevant when crops lack a shared evolutionary history with the invading pathogen (Singh et al., 2023).

Fungal pathogens are among the most destructive phytopathogens worldwide and are estimated to account for approximately 80% of crop diseases, causing severe yield losses, particularly in horticultural systems. Among them, *Fusarium oxysporum* is one of the most widespread soil-borne pathogens, affecting a broad range of economically important crops, including tomato, banana, cucumber, watermelon, muskmelon, cotton and legumes. *F. oxysporum* persists in soil and plant debris for long periods, and causes vascular wilt and root rot throughout all stages of plant development, often leading to plant death (Attia et al., 2022; Boulahouat et al., 2023; Yan et al., 2023).

Chemical fungicides remain the primary strategy for disease management in agricultural systems. However, the intensive and prolonged reliance on synthetic products has raised increasing concerns for environmental sustainability, ecosystem integrity, and human health. Their extensive use has been associated with soil and water contamination, alterations of beneficial soil microbial communities, and the progressive emergence of resistant plant pathogens (Tang et al., 2025). These challenges have stimulated growing interest in alternative and more sustainable disease management strategies, including the use of microbial biological control agents (MBCAs) (Giorni et al., 2024; Modiba et al., 2025; Tyagi et al., 2024).

Within the MBCA category, fluorescent pseudomonads have garnered significant attention due to their broad physiological plasticity and ability to thrive across different plant-associated environments. Their pronounced ecological fitness enables efficient establishment and persistence in multiple niches, including the rhizosphere, phyllosphere, and internal plant tissues, where they effectively compete with other microorganisms for space and nutrients. Such competitive abilities constitute an important component of their biocontrol efficacy (Bonaterra et al., 2022; Vicentini et al., 2022).

An additional key mechanism underlying the biocontrol activity of fluorescent pseudomonads is their capacity to produce a wide range of bioactive secondary metabolites, including 2,4-diacetylphloroglucinol (DAPG), hydrogen cyanide (HCN), phenazines (PHZ), pyrrolnitrin (PRN), pyoluteorin (PLT), cyclic lipopeptides (CLPs), lytic enzymes, and siderophores such as pyoverdine. Collectively, these compounds contribute to antagonism against phytopathogens (Maurya et al., 2024; Mishra and Arora, 2018). Moreover, some fluorescent pseudomonads can activate plant defense responses through induced systemic resistance (ISR), further enhancing host protection against a broad spectrum of pathogens (Vicentini et al., 2022).

A critical aspect in the effective use of microbial biocontrol agents (MBCAs) is a clear understanding of the molecular mechanisms underlying their antifungal activity and its regulation. In particular, deciphering the transcriptional programs activated during interactions between MBCAs and phytopathogenic fungi is essential to explain biocontrol efficacy.

Previous transcriptomic works examined the transcriptional response of *Pseudomonas* strains during fungal interaction. In one study by Hennessy et al. (2017), fungal exposure induced a selective reorganization of gene expression of *P. fluorescens*, characterized by strong upregulation of genes involved in secondary metabolite detoxification and metabolism. Additional transcriptomic analyses on other pseudomonads have shown that genes involved in antimicrobial metabolite biosynthesis, such as HCN and phenazine-related compounds, as well as siderophore production and alginate biosynthesis, were actively transcribed during fungal interaction (Dai et al., 2024; Nelkner et al., 2019).

The aim of this study was to investigate the molecular basis of antifungal activity in the MBCA strain *Pseudomonas* sp. PF05 by combining comparative transcriptomic and genomic analyses under two complementary conditions. First, we compared both the genomic content and the basal transcriptional profile of PF05 with those of *Pseudomonas frederiksbergensis* PF4.89, a closely related strain with markedly reduced biocontrol activity, to assess whether their contrasting phenotypes are primarily associated with differences in gene repertoire, strain-specific regulation of shared genes, or a combination of both. Second, we compared the gene expression profile of PF05 grown in the presence of *F. oxysporum* with that of PF05 grown in its absence, in order to identify genes specifically up- or down-regulated during bacterial-fungal interaction. The selection of the two strains *Pseudomonas* sp. PF05 and *P. frederiksbergensis* PF4.89 was based on previous phenotypic evidence of markedly different biocontrol performance. In a previous study in which multiple *Pseudomonas* strains were tested against different phytopathogenic fungi, PF05 exhibited the highest antifungal activity, particularly against *F. oxysporum* (84.16% inhibition), whereas PF4.89 showed a substantially lower inhibitory effect (32.07%) under identical experimental conditions (Di Rico et al., 2025).

By integrating comparative genomics with transcriptomic profiling, we evaluated whether genes unique to PF05 were transcriptionally active under the tested conditions and whether conserved genes displayed strain-specific regulatory patterns. This combined approach enabled the identification of genetic determinants whose contribution to biocontrol may depend on genomic content (PF05-specific genes) and/or regulatory differences affecting shared functional pathways. Overall, this study contributes to efforts to improve MBCA reliability by clarifying the molecular basis of antifungal activity against *F. oxysporum*. Such knowledge can support the identification of genetic markers for strain selection and help guide the development of more effective and predictable biocontrol candidates for sustainable disease management.

## 2 Materials and methods

### 2.1 Genomic analyses

#### 2.1.1 Genome sequencing, assembly, and annotation

To obtain good quality genomes, both strains were cultivated by transferring a single colony from a freshly grown plate into 10 mL Falcon tubes containing 5 mL of tryptic soy broth (TSB) and incubated overnight at 30 °C with shaking at 180 rpm. Genomic DNA (gDNA) was extracted using the E.Z.N.A.® Bacterial DNA Kit (Omega Bio-Tek, USA) according to the manufacturer’s instructions. DNA quality and integrity were verified by electrophoresis on a 1% (w/v) agarose gel and quantified using a NanoDrop spectrophotometer (Thermo Fisher Scientific, Waltham, MA, USA).

Whole-genome sequencing was performed using the Illumina NovaSeq 6000 platform (Novogene, Cambridge, United Kingdom). Raw reads were quality-filtered by removing sequences containing more than 50% low-quality bases (*Q* ≤ 5) and trimmed to remove adapters and residual low-quality regions prior to assembly. Genome assembly was performed *de novo* with Unicycler v0.5.1 (Wick et al., 2017) using default parameters, discarding contigs with low coverage. Assembly quality was evaluated using QUAST v5.3.0 (Gurevich et al., 2013) and CheckM v2 (Parks et al., 2015).

Taxonomic identification of the two strains was performed through a stepwise genome-based workflow. First, both the assembled genome (FASTA) and the raw reads (FASTQ) were submitted to the Similar Genome Finder tool available on the BV-BRC platform (Wattam et al., 2024), which applies the Mash/MinHash algorithm (Ondov et al., 2016) to rapidly identify the closest reference genomes based on shared k-mer content. The candidate species showing the highest similarity scores were then evaluated using the Type Strain Genome Server (TYGS), which performs *in silico* digital DNA–DNA hybridization (dDDH) and infers an intergenomic distance-based phylogenomic tree with branch support using FASTME 2.1.6.1 (Lefort et al., 2015).

The top-scoring species was subsequently validated using the ANI calculator in the EzBioCloud platform to compute pairwise Average Nucleotide Identity (ANI%) (Yoon et al., 2017).

Species delineation followed widely accepted thresholds of ≥70% dDDH and ≥95% ANI. Functional annotation of the final assemblies was performed with Bakta v1.9.3, which provides standardized, taxonomy-independent annotations derived from UniProt’s UniRef protein cluster database (Schwengers et al., 2021).

#### 2.1.2 Whole genome analyses and comparisons

Whole-genome comparison between PF4.89 and PF05 was performed to identify genomic determinants potentially associated with their contrasting biocontrol phenotypes. Annotated protein sequences generated by Bakta were uploaded to the PGPT-Pred tool available on PLaBAse platform for the prediction of biocontrol related traits (Patz et al., 2021).

The analysis was carried out using the strict mode, which integrates BLASTp and HMMER-based searches to detect PGPT-associated protein families with high confidence. The PLaBAse workflow allowed the identification of functional categories related to gene clusters and protein families associated with biocontrol activity, including pathways linked to the synthesis of phenazine derivates, bactericidal and fungicidal compounds, volatiles, and siderophores.

### 2.2 Transcriptomic analyses

The transcriptomic experimental design included three different biological conditions: (i) *Pseudomonas* sp. PF05 grown in dual culture plate with *F. oxysporum* (hereafter referred to as PF05_DP), (ii) PF05 grown in monoculture without the fungus (PF05), and (iii) *P. frederiksbergensis* PF4.89 grown in monoculture (PF4.89). Two main transcriptomic comparisons were performed. The comparison between PF05_DP and PF05 was performed to investigate the transcriptional response of PF05 to fungal interaction. In parallel, the comparison between PF4.89 and PF05 under basal (fungus-free) conditions aimed to identify transcriptional differences between the two strains. For each condition, three independent biological replicates were prepared. A limitation of this experimental set up is that transcriptomic profiling during fungal interaction was performed only for PF05. Therefore, strain-specific differential expression under interaction conditions (PF05_DP vs. PF4.89_DP) are not assessed.

#### 2.2.1 RNA extraction

Dual-culture and monoculture plates were prepared following the procedure described by Di Rico et al. (2025), with minor adaptations. All experimental conditions were carried out on PDA agar plates. For the dual-culture condition (PF05_DP), plates were inoculated at the center with a mycelial plug (9 mm) excised from the actively growing margin of an *F. oxysporum* culture. A standardized bacterial suspension of *Pseudomonas* sp. PF05 (10^8^ CFU/mL) was streaked as two parallel lines on opposite sides of the fungal plug, at an approximate distance of 25 mm. For monoculture assays, PF05 and PF4.89 were streaked separately on PDA plates under the same experimental conditions. Plates were incubated for 3 days at 25 °C. Following incubation, bacterial biomass was collected for RNA extraction. Specifically, half of each bacterial streak in every replicate (PF05_DP, PF05, and PF4.89), corresponding to the portion facing the center of the plate, was harvested using a sterile loop and immediately resuspended in a stabilization solution consisting of two volumes of RNAprotect® Bacteria Reagent (Qiagen, Hilden, Germany) and 1 volume of TSB. After incubation of 5 min at room temperature, suspensions were centrifuged for 10 min at 5,000×*g*, and the resulting pellets processed for RNA extraction.

Total RNA was isolated and purified using the RNeasy Mini Kit (Qiagen, Hilden, Germany), following the manufacturer’s protocol for Gram-negative bacteria, with modifications to enhance cell lysis. Briefly, bacterial pellets were resuspended in 200 μL of TE buffer containing 15 mg mL^−1^ lysozyme and incubated at 37 °C with shaking (700 rpm) for 2 h. Subsequently, 10 μL of TE buffer containing 20 mg mL^−1^ proteinase K was added, followed by an additional incubation at 37 °C for 1 h under the same conditions. RNA purification was then completed according to the standard protocol, including on-column DNase treatment using the QIAGEN RNase-Free DNase Set to remove residual genomic DNA.

RNA quantity and quality were assessed using an Agilent TapeStation 4150 system (Agilent Technologies, Santa Clara, CA, USA). Only RNA samples with RNA Integrity Number equivalent (RINe) values above 6.0 were considered suitable for downstream analyses, and all samples used in this study displayed RINe values between 8.0 and 9.0. In addition, RNA integrity was preliminarily verified by electrophoresis on a 2% agarose gel to confirm the absence of RNA degradation.

#### 2.2.2 RNA sequencing

RNA sequencing and data analyses were performed by Novogene Co (Cambridge, United Kingdom). Total RNA was first depleted of ribosomal RNA and then precipitated with ethanol. The remaining RNA was fragmented, and the first-strand cDNA was synthesized using random hexamer primers. To ensure strand specificity, dUTPs were incorporated in place of dTTPs during the second-strand synthesis, and the resulting cDNA underwent end repair, A-tailing, adapter ligation and size selection. PCR amplification was subsequently carried out, during which the polymerase selectively excluded the strand containing dUTPs, thereby preserving transcript orientation. Following a final purification step, a directional cDNA library was obtained. Library concentration was measured using Qubit fluorometry (Invitrogen Life, Thermo Fisher Scientific, Waltham, MA, USA) and real-time PCR, while fragment size distribution was assessed with a bioanalyzer. After quality control, libraries were pooled according to their effective concentration and the required data output, and finally sequenced on Illumina platforms.

#### 2.2.3 RNA-Seq alignment and expression quantification

Raw sequencing reads in FASTQ format were processed using fastp to remove adapter contamination, poly-N sequences and low-quality reads, resulting in a high-confidence dataset of clean reads. Parameters such as Q20, Q30 and GC content were then calculated to evaluate sequencing quality and error rates. Subsequently, clean reads were aligned to the reference genomes of *Pseudomonas* sp. PF05 and *P. frederikbergensis* PF4.89 using Bowtie2 (v2.5.4). Gene-level quantification was performed with featureCounts (v2.0.6), generating the read counts mapped to each gene. Expression values were then normalized as FPKM (Fragments Per Kilobase of transcript per Million mapped reads), which adjusts for gene length and sequencing depth, thereby enabling reliable comparisons of gene expression levels across and within samples.

#### 2.2.4 Differential expression analyses

Differential gene expression analyses were performed using the DESeq2 R package (v1.42.0), which applies statistical models based on the negative binomial distribution to identify significantly regulated genes between conditions. *p*-values were corrected using the Benjamini-Hochberg method to control the false discovery rate (FDR). Genes with an adjusted *p*-value (padj) ≤ 0.05 and |log_2_ (fold change)| ≥ 1 were considered significantly differentially expressed (DEGs).

Gene Ontology (GO) and KEGG pathways enrichment analyses of DEGs were performed using the clusterProfiler R package (v4.8.1), with correction for gene length bias. GO terms and KEGG pathways with a padj ≤ 0.05 were considered significantly enriched. In parallel, Gene Set Enrichment Analysis (GSEA) was carried out to assess whether predefined gene sets from GO and KEGG showed coordinated expression changes between the two conditions PF05_DP and PF05. All genes were ranked according to their log_2_ fold change, including those not significantly differentially expressed, and enrichment was evaluated based on their distribution along the ranked list. GSEA was performed locally using the Broad Institute software,^1^ with GO and KEGG datasets analyzed independently.

#### 2.2.5 Identification of orthologous genes for RNA-seq comparison

Shared genes between the two strains were identified as putative orthologues using a bidirectional (reciprocal) BLAST approach. Sequence datasets derived from each strain, namely unigene sequences and EST datasets, were used alternately as query and database in reciprocal BLAST searches. Orthologous pairs were defined based on reciprocal best-hit criteria and BLAST similarity parameters.

## 3 Results

### 3.1 Comparative genomic analyses

**Figure 1:**
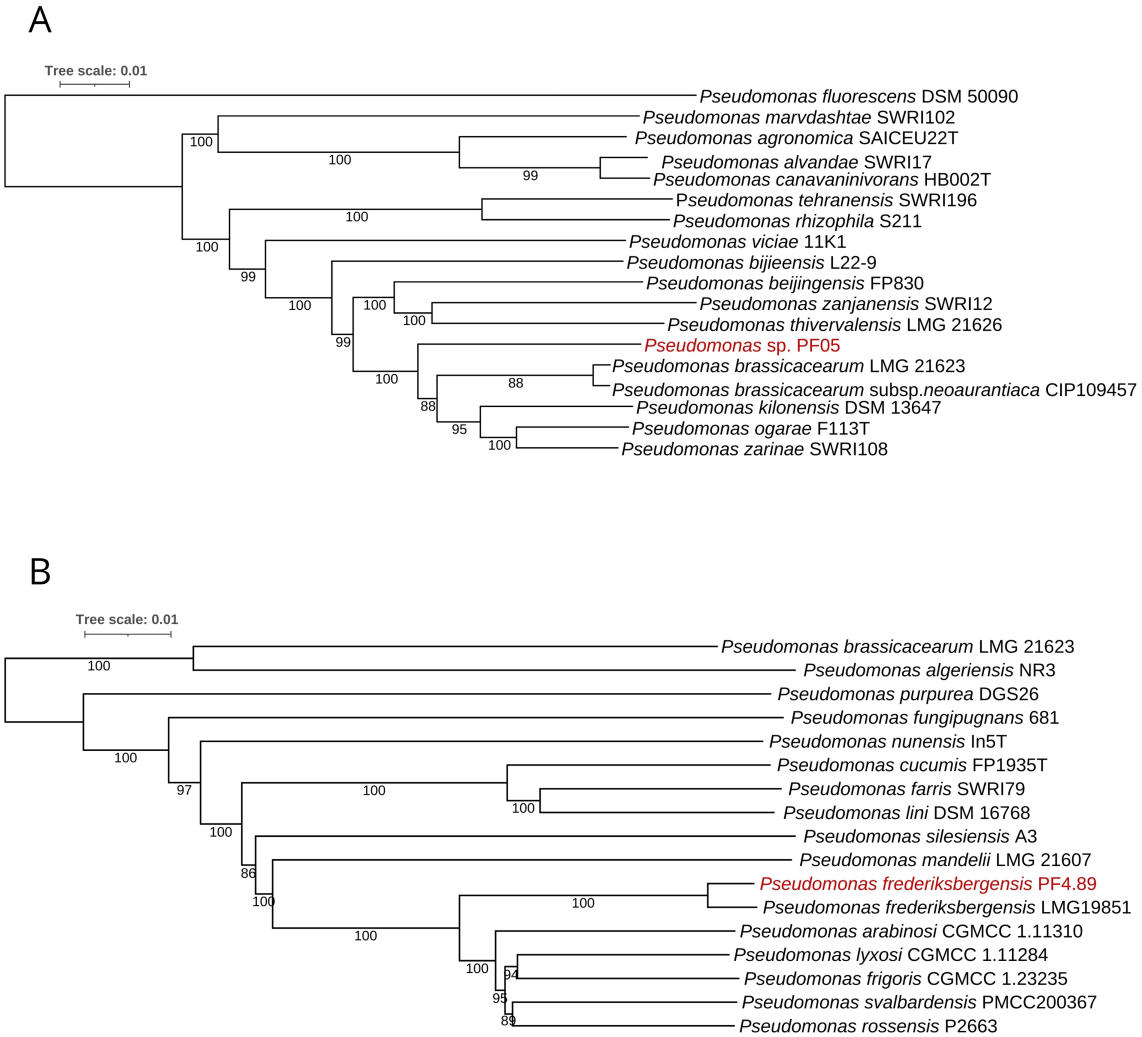
Midpoint-rooted phylogenetic trees of *Pseudomonas* sp. PF05 **(A)** and *P. frederiksbergensis* PF4.89 **(B)** compared with their respective closely related *Pseudomonas* type strains. Branch support was estimated from 100 bootstrap replicates.

Taxonomic assignment based on whole-genome relatedness showed contrasting levels of species resolution between the two strains. For PF05, the highest dDDH value obtained through TYGS was 56.9% when compared with the closest type strain *P. brassicacearum* subsp. *neoaurantiaca* CIP 109457, which falls below the accepted species demarcation threshold of 70%. This result was consistent with the ANI analysis, which yielded 94.14%, also below the ≥95% boundary required for species-level classification. Together, these metrics indicate that PF05 cannot be confidently assigned to any currently described species (Figure 1A). In contrast, PF4.89 displayed a highest dDDH value of 90.7% when compared with the type strain *P. frederiksbergensis* LMG 19851, and this identification was further supported by the ANI value of 98.83% against the same reference genome. These results indicate that PF4.89 is a member of the species *P. frederiksbergensis* (Figure 1B).

Both genomes were subsequently screened for PGPTs using the PLaBAse strict-mode pipeline. Several PGPT-associated genes were identified in both strains, although some differences were detected. In PF05, 25% of the PGPT hits were related to plant colonization, while 21% were associated with competitive exclusion and a further 21% with stress control and biocontrol functions. Additional PGPTs were linked to bio-fertilization (13%), phytohormone and plant signal production (10%), and bio-remediation (8.0%) (Supplementary Table S1). PF4.89 displayed a similar distribution across general PGPT categories (Supplementary Table S2), but the number and type of hits associated with biocontrol-specific functions differed substantially. In *Pseudomonas* sp. PF05, bactericidal compound-associated genes represented 2.15% of the total PGPT hits, followed by genes related to volatile compound production (1.25%), fungicidal compounds (0.56%), phenazine derivatives (0.29%), and polyketides (0.13%). In *P. frederiksbergensis* PF4.89, bactericidal compound-associated genes accounted for 1.97% of PGPT hits, while genes related to volatile compounds represented 1.38%. Lower proportions were observed for fungicidal compounds (0.40%), phenazine derivatives (0.20%), and polyketides (0.04%).

Overall, PF05 displayed higher relative proportions of genes associated with fungicidal compounds, phenazine derivatives, and polyketide biosynthesis compared with PF4.89, whereas both strains showed comparable representation of bactericidal- and volatile compound-associated functions (Table 1). At the gene level, PF05 contained a more complete phenazine-associated repertoire, including *phzF* and *pqsH*, whereas PF4.89 lacked *pqsH* and exhibited a reduced phenazine-related gene set. In addition, PF05 displayed a broader complement of polyketide-associated genes (*actIV*, *actV*A6, *oxyN*, *oxyL*) compared with PF4.89, which retained a more limited subset (*actIV*, *otrA*).

**Table 1:** Secondary metabolite–related PGPTs associated with biocontrol functions in *Pseudomonas* sp. PF05 and *P. frederiksbergensis* PF4.89, identified using the PLaBAse pipeline.

Strain	Bactericidal compounds 2.15% ^1^	Volatile compounds 1.25%	Fungicidal compounds 0.56%	Phenazine derivates 0.29%	Polyketides 0.13%
*Pseudomonas* sp. PF05	*tktAB, acpP, auaH, pksJ, cvpA, rseP, yadG, ecsB, ycgN, dcsE, ddaF, lgrE, aaeA, pcaK, ubiC, fcbC1, nocI, cefD, pmrAB, fabD, fabI, fabG, pltP, plti, yejA, yejB, yejE, yejF, ABC_SP_A, ABC_SP_P1, ABC_SP_P, ABC_SP_S, gsp, paiA, potF, potG, potH, potI, putE, puuP, speA, speC, speE, speG, spuC, phoP, phoQ, syrB1, sypA, ribA, ribD, oprM, toxJ, tfuA_like, tycC, cepH*	*aceF-pdhC, acoABC, acoR, acuC, budB-ilvK-alsS-ilvB-ilvG-ilvI, budC, butB, ilvHN, lpd-pdhD, adh1, bdh, hmgL, scoAB, yghZ, ubiC, accABCD, acd, fabD-bmyD, fabF, fabH, fabI, fabZ, fadD, fadN, fabG, hcnABC*	*chbG,* chitin_deacetylase*, nagZ, epoD, ppsD-fenA, adiA, lysS, speA, speC, yajQ, ybgC, plpE,* Membrane_bound_beta_hydroxylase, Putative_transcription_factor, *thaA, cmaB, thaC2, thaD, ribA, ribD, toxI-oprM*	*aroAB, aroEFGH, aroKL, aroQ,* prephenate_dehydrogenase*, phzF, pqsH, phnAB*	*actIV, actVA6, oxyN, otrA, oxyL*

Strain	Bactericidal compounds 1.97%	Volatile compound 1.38%	Fungicidal compounds 0.40%	Phenazine derivates 0.20%	Polyketides 0.04%
*P. frederiksbergensis* PF4.89	*tktAB, phnW, acpP, auaH, cvpA, nisT, rseP, ybhF, ycgN, dcsE, ddaF, aaeA, pcaK, ubiC, ybgC, pikC, cefD, pmrA, pmrB, fabD, fabI, redL, ymfI, pltR, plti, yejA, yejB, yejE, yejF, ABC_SP_A, ABC_SP_P1, ABC_SP_P, ABC_SP_S, paiA, potA, potB, potC, potF, potG, potH, potI, putE, puuP, speABC, speE, speG, spuC, phoP, phoQ, ribA, ribD, toxF, toxI, cepH*	*aceF, acoAB, acoR, acuC, ilvBIGK, budC, butB, ilvHN, lpd, adh1, bdh, hmgL, scoAB, yghZ, ubiC, accABCD, acd, fabDB, fabFGHI, fabZ, fadD, fadN, hcnB*	*asm17,* chitin_deacetylase*, nagZ, adiA, lysS, speA, speC, yajQ, ybgC,* putative_hydrolase*, ribA, ribD, toxI-oprM*	*aroAB, aroEF, aroKL, aroQ,* prephenate_dehydrogenase*, phnAB*	*actIV, otrA*

Genes involved in bactericidal compounds, volatile compounds, fungicidal compounds, phenazine derivatives, and polyketides are reported. Percentages refer to the contribution of each category to the total PGPT repertoire of each genome.

^1^Percentages reported for each PGPT group represent the proportion of genes identified within that functional category relative to the total number of PGPT hits detected across all genomes using the PLaBAse database.

Genes linked to non-ribosomal peptide (NRP) or lipopeptide-associated pathways (*syrB*1, *sypA*, *tycC*, *tfuA*-like) were detected exclusively in PF05. Both strains harbored genes involved in chitin and cell-wall related processes; however, PF05 additionally possessed genes associated with secondary metabolite maturation and polymeric substrate interaction (*ppsD*–*fenA*, *thaA*/*thaC*2/*thaD*, *cmaB*). Furthermore, PF05 encoded a more extensive set of genes involved in secondary metabolite biosynthesis and regulation, including components of NRPS-PKS-associated clusters (e.g., *redL*, *cefD*, *pikC*) and a complete HCN synthase operon (*hcnABC*), whereas PF4.89 retained only partial representations of these pathways.

Both strains carried genes related to polyamine metabolism, two-component regulatory systems, and efflux mechanisms; however, PF05 exhibited a broader representation of these functions, consistent with an expanded genetic repertoire associated with secondary metabolite production, regulation, and persistence under competitive conditions. Together, these differences indicate an expanded and more diverse secondary metabolite gene repertoire in PF05 relative to PF4.89.

### 3.2 Transcriptomic responses associated with fungal interaction and strain-specific regulation

To investigate transcriptional changes associated with fungal interaction and strain-specific regulatory differences, two comparative RNA-seq analyses were performed. The transcriptional response of *Pseudomonas* sp. PF05 to *F. oxysporum* was assessed by comparing dual-culture (PF05_DP) and monoculture (PF05) conditions. In parallel, basal transcriptional differences were examined between PF05 and *P. frederiksbergensis* PF4.89 grown in the absence of the fungus, aiming to identify regulatory features associated with contrasting biocontrol phenotypes.

#### 3.2.1 Transcriptional response of *Pseudomonas* sp. PF05 to *Fusarium oxysporum*

**Figure 2:**
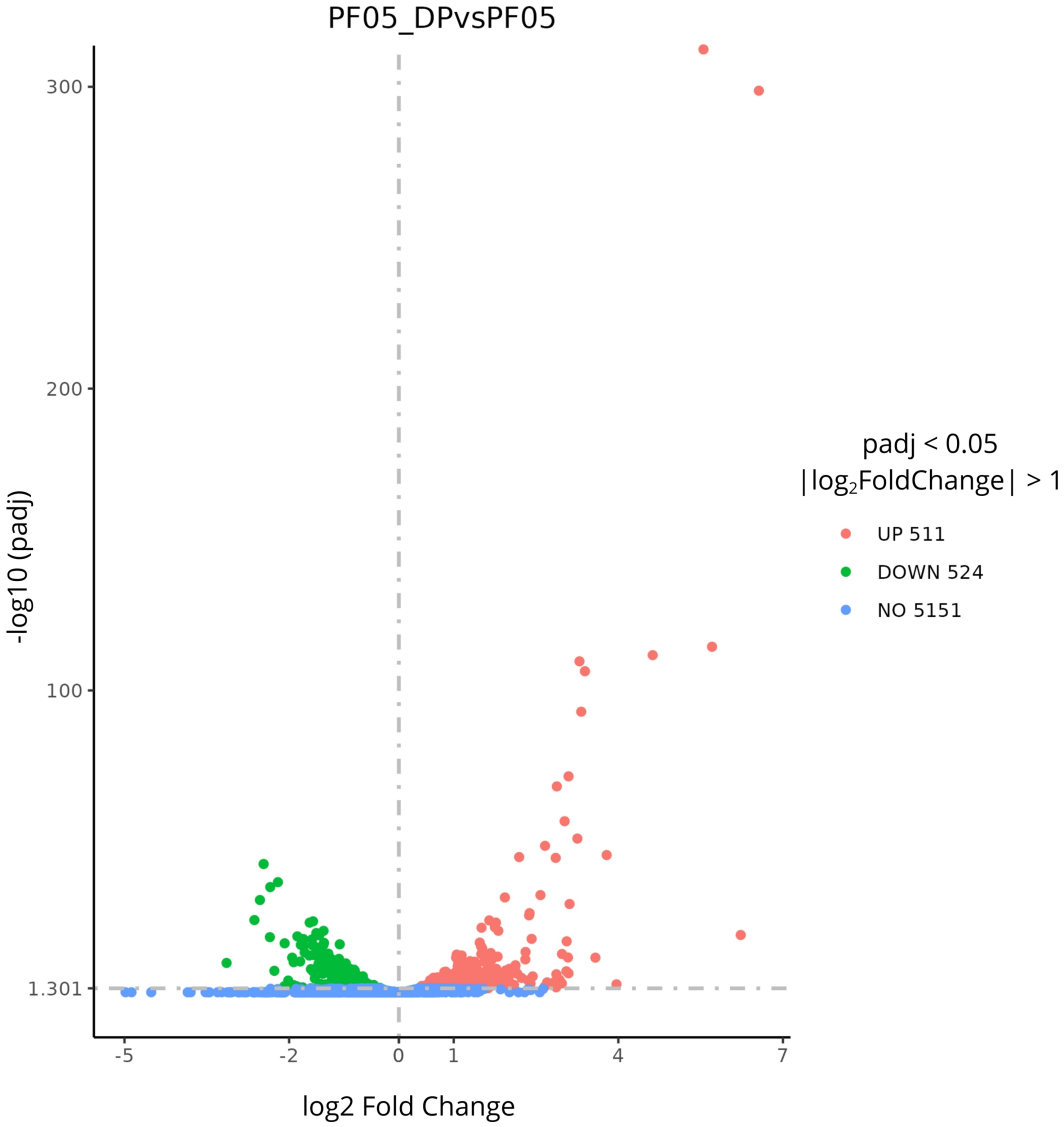
Volcano plot of DEGs between *Pseudomonas* sp. PF05_DP and *Pseudomonas* sp. PF05. The *x*-axis displays the log_2_ fold change and the *y*-axis the -log10 adjusted *p*-value. Significantly up-regulated genes are shown in red, down-regulated in green and non-significant genes in blue.

High-quality RNA-seq data were obtained for all samples, with consistently high mapping rates and comparable GC content across conditions. After filtration of the raw reads (~16–19 million per sample), ~15.5–18.7 million clean reads were retained across the replicates of both conditions, indicating a negligible read loss compared with the raw data. Error rates (0.01%) as well as Q20 (>98%) and Q30 (>95%) values were comparable, confirming the overall reliability of the sequencing process. The total mapping rate reached ~94% for PF05 and ~91% for PF05_DP. Global gene expression profiles were also largely overlapping between PF05 and PF05_DP. Most genes showed moderate to high expression levels, with ~29–39% of genes falling in the 3–15 FPKM range and ~34–38% clustering between 15 and 60 FPKM, which represented the dominant expression class under both conditions. Only a small fraction of genes (0.5–6%) exhibited very low expression (0–1 FPKM), whereas 18–21% were highly expressed (>60 FPKM). Global expression profiles were largely conserved between PF05 and PF05_DP, indicating that fungal interaction induces targeted transcriptional reprogramming rather than widespread expression shifts. Differential expression analysis identified 1,035 differentially expressed genes (DEGs) between PF05_DP and PF05 (|log_2_FC| ≥ 1, adjusted *p*-value < 0.05), including 511 up-regulated and 524 down-regulated genes, while 5,151 genes showed no significant change in expression (Figure 2).

#### 3.2.2 Functional categories of genes up- and down-regulated during fungal interaction

Supplementary Table S3 reports the full list of genes found to be equally expressed, up- or down-regulated in *Pseudomonas* sp. PF05_DP compared with PF05, ordered by increasing *p*-value and adjusted *p*-value. After assigning a Gene Ontology–based function to each gene, the most represented up-regulated DEGs were organized into three main macro categories that reflect their biological role: (i) membrane transport and efflux, (ii) metabolism and (iii) regulation, as shown in Table 2. The metabolic category was further divided into redox metabolism and small molecule metabolism, and only genes displaying the highest log_2_ fold change values were included in each subgroup.

**Table 2:** Functional classification of the most represented up-regulated genes in *Pseudomonas* sp. PF05_DP compared with PF05.

Up-regulated DEGs - *P. fluorescens* PF05_DP vs PF05			
	Gene name	log_2_FC	padj
Transmembrane transport and efflux	Outer membrane protein H1	5.546664843	0
	Fusaric acid resistance domain protein	6.561549078	1.99E−299
	Urea ABC transporter substrate-binding protein	2.420336456	2.05E−18
	Ammonia channel protein AmtB	1.502630945	2.19E−13
	Major facilitator superfamily (MFS) profile domain-containing protein	2.305382908	1.32E−11
	Cyanate transporter%2C major facilitator family	1.138776192	1.37E−11
	ABC transmembrane type-1 domain-containing protein	1.276580988	1.58E−10
	Sugar phosphate permease	2.117717051	7.73E−09
	Permeases of the major facilitator superfamily	1.021786137	7.95E−07
	Outer membrane efflux protein BepC	1.377762261	8.78E−07
	Ammonium transporter AmtB-like domain-containing protein	1.3300965	4.03E−06
	RND efflux pump membrane fusion protein barrel-sandwich domain-containing protein	0.789193861	1.06E−05
	Various polyols ABC transporter%2C permease component 1	1.601244107	1.11E−05
	Multidrug efflux pump subunit AcrB	0.929080124	0.00014225
	Choline ABC transporter permease subunit	1.347970967	0.000185027
	Channel protein TolC	0.752849604	0.000360507
	ABC-type glycine betaine transport system substrate-binding domain-containing protein	2.82643374	0.000568948
	Sugar ABC transporter permease	1.409638345	0.000744494
	Small-conductance mechanosensitive channel	1.02754723	0.002207278
	Urea ABC transporter permease subunit UrtC	1.954495568	0.00493599
	Nitrate/nitrite transporter	1.844233513	0.006875514
	ABC-type dipeptide/oligopeptide/nickel transport system%2C permease component	0.996607035	0.011535849
Redox metabolism	Protein-methionine-sulfoxide reductase catalytic subunit MsrP	2.879568286	6.25E−69
	Protein-methionine-sulfoxide reductase heme-binding subunit MsrQ	3.253742015	1.18E−51
	4-Hydroxybenzoate 3-monooxygenase	2.580881902	6.26E−33
	Quercetin 2%2C3-dioxygenase	3.111314054	5.84E−30
	GMC family oxidoreductase	1.750060756	2.72E−22
	(R%2CR)-butanediol dehydrogenase/meso-butanediol dehydrogenase/diacetyl reductase	3.058584967	1.35E−17
	4-(Hydroxymethyl)benzenesulfonate dehydrogenase TsaD1	2.309510091	4.39E−14
	FMN dependent NADH: quinone oxidoreductase	2.973372679	2.13E−13
	Putative quinone oxidoreductase	1.670650635	2.94E−13
	Acyl-CoA reductase or other NAD-dependent aldehyde dehydrogenase	3.08234434	2.96E−12
	Acetoin dehydrogenase E1 component%2C beta subunit	3.581728501	3.41E−12
	4-Hydroxyphenylpyruvate dioxygenase	1.129441372	5.86E−11
	Glycine/D-amino acid oxidase (deaminating)	2.012666504	1.78E−08
	Glutathione s-transferase family protein	1.048113256	1.38E−07
	Putative dioxygenase of extradiol dioxygenase family	1.540046926	2.89E−07
	Sarcosine oxidase%2C delta subunit	2.870983267	1.08E−06
	Carboxymuconolactone decarboxylase-like domain-containing protein	2.003024328	7.00E−06
	Cytochrome c domain-containing protein	1.523561059	7.26E−06
	Acetoin dehydrogenase E1 component%2C alpha subunit	2.971565702	0.001179217
	Dimethylglycine demethylation protein DgcA	2.098955636	0.003353236
	NADPH-dependent 2%2C4-dienoyl-CoA reductase%2C sulfur reductase%2C or a related oxidoreductase	1.701764862	0.006395679
	Nitrite reductase [NAD(P)H]%2C small subunit	2.037848483	0.02754759
Small-molecule metabolism	Glutamate--ammonia ligase	1.506899799	4.14E−22
	Fumarylacetoacetase	1.47372097	3.49E−17
	Ethanolamine ammonia-lyase%2C large subunit	1.623377025	1.37E−11
	Urease accessory protein UreE	3.052962725	1.16E−07
	Urease subunit alpha	1.535742186	6.99E−07
	Ethanolamine ammonia-lyase subunit EutC	1.160754409	9.67E−07
	Urease subunit beta	2.439708301	5.49E−06
	Urease subunit gamma	1.786207645	2.32E−05
	Acetoin catabolism protein	3.966445151	0.002269745
Regulation	Virulence transcriptional regulatory protein PhoP	4.626707637	2.01E−112
	Transcriptional regulator%2C GntR family	1.769304713	8.06E−24
	Nitrogen regulation protein NR(I)	1.555614736	6.97E−11
	HTH luxR-type domain-containing protein	1.380065721	7.33E−11
	HTH gntR-type domain-containing protein	1.417047088	6.77E−10
	HTH-type transcriptional regulator CysL	1.072564064	2.04E−09
	DNA-binding response regulator%2C LuxR family	0.908345692	1.98E−05
	AcoR	0.898973707	5.55E−05
	HTH crp-type domain-containing protein	0.918206382	0.0006357
	Transcriptional regulator GcdR	0.893024171	0.00183556
	LuxR family transcriptional regulator	0.817882504	0.002915036

Up-regulated DEGs were grouped into functional categories based on Gene Ontology annotations, and the most represented biological categories were identified. Within each category, genes showing the strongest up-regulation are reported together with their log_2_FC and adjusted *p*-values.

Within the transport and efflux group, several ABC transporters and members of the Major Facilitator Superfamily (MFS) were up-regulated, together with membrane proteins responsible for the extrusion of toxic compounds. Among these, a fusaric acid resistance domain protein, the outer membrane protein H1 and components associated with RND-family multidrug efflux systems were identified. Notably, the fusaric acid resistance domain protein and outer membrane protein H1 were the most strongly induced genes in this category, and across the entire dataset, showing log_2_ fold changes of 6.56 and 5.55, respectively.

Among up-regulated genes involved in metabolic processes, the redox metabolism subgroup comprised oxidoreductases contributing to intracellular redox balance, neutralization of reactive oxygen species and detoxification pathways. Highly induced genes encoded the MsrPQ methionine sulfoxide reductase system, quercetin 2,3-dioxygenase, acetoin and butanediol dehydrogenases, and an acyl-CoA reductase. Genes assigned to small molecule metabolism were mainly involved in the synthesis and catabolism of organic acids and nitrogen-containing compounds. In this subgroup, the most strongly overexpressed genes encoded the various components of urease and the protein responsible for acetoin catabolism. With respect to transcriptional regulation, several up-regulated genes belonged to the LuxR family and played a key role in quorum sensing. The most induced regulatory gene was the virulence transcriptional regulatory protein PhoP, which exhibited a log_2_ fold change of 4.62.

Following functional annotation, the most represented down regulated DEGs were grouped into two main macro categories, namely (i) transport and secretion and (ii) aerobic and anaerobic respiration, as shown in Table 3, which includes only genes with the lowest log_2_ fold change values. Within the transport and secretion category, multiple ABC transporters were down-regulated, together with MFS proteins, permeases, and subunits of the tripartite tricarboxylate transporters (TTT), as well as components of the type II and type VI secretion systems. Marked repression was observed for genes involved in iron uptake, including an iron permease and a periplasmic iron-binding lipoprotein.

**Table 3:** Functional classification of the most represented down-regulated genes in *Pseudomonas* sp. PF05_DP compared with PF05.

Down-regulated DEGs - *P. fluorescens* PF05_DP vs PF05			
	Gene name	log_2_FC	padj
Transport and secretion	Iron permease%2C FTR1 family	−2.203826554	3.33E−37
	Periplasmic lipoprotein involved in iron transport	−2.343902186	1.43E−35
	Tripartite-type tricarboxylate transporter%2C extracytoplasmic receptor component TctC	−2.350377636	5.52E−19
	Mechanosensitive ion channel family protein	−1.580557693	3.03E−18
	ABC-2 type transporter domain-containing protein	−1.631592889	6.76E−13
	ABC transmembrane type-2 domain-containing protein	−1.396474305	1.16E−08
	ABC transporter permease	−1.1775995	5.98E−08
	Amino acid transporter transmembrane domain-containing protein	−1.564839641	1.16E−07
	Cytosine permease	−1.235340589	8.28E−07
	ABC-type polar amino acid transport system%2C ATPase component	−0.923103904	1.20E−06
	Major facilitator superfamily (MFS) profile domain-containing protein	−0.966857141	3.77E−06
	Type IV pilus biogenesis protein PilQ	−0.870058819	2.71E−05
	ABC transporter domain-containing protein	−0.891062531	6.81E−05
	ABC-type glycine betaine transport system substrate-binding domain-containing protein	−0.876127219	9.16E−05
	HlyD family efflux transporter periplasmic adaptor subunit	−0.923270763	0.000100768
	Glycine betaine transport ATP-binding protein OpuAA	−0.882090583	0.000268854
	RCK N-terminal domain-containing protein	−0.783475677	0.000538529
	Type VI secretion system tubule-forming protein VipA	−0.841105013	0.00066635
	ABC transmembrane type-1 domain-containing protein	−0.797463808	0.000848119
	Ribose ABC transporter%2C permease protein	−0.887114307	0.000879443
	Type VI secretion system baseplate subunit TssK	−0.695629405	0.001005678
	MFS transporter	−0.750158023	0.001141814
	Phosphate ABC transporter ATP-binding protein PstB	−0.83496613	0.001355109
	Porin	−1.148240798	0.002822793
	Tripartite tricarboxylate transporter TctB family protein	−2.087591805	0.010174548
	Efflux pump membrane transporter	−1.168373989	0.011834568
	Type II secretion system protein H	−0.739009337	0.01905778
	Type II secretion system secretin GspD	−1.823634185	0.021115968
	ABC transporter in pyoverdin gene cluster%2C ATP-binding component	−0.720908548	0.031127907
	Type VI secretion system membrane subunit TssM	−0.680081954	0.035282683
	Gluconate permease	−1.012474496	0.041722858
Aerobic and anaerobic respiration	4Fe-4S ferredoxin-type domain-containing protein	−2.464150445	3.23E−43
	Cytochrome C oxidase	−2.081038373	6.06E−17
	NosF	−1.785906814	1.93E−16
	Aspartate ammonia-lyase	−1.570158729	2.98E−16
	NosL	−1.734150888	3.24E−16
	Cytochrome c oxidase accessory protein CcoG	−1.497060353	1.35E−15
	Nitrous oxide reductase family maturation protein NosD	−1.392120372	1.66E−13
	NirN	−1.55188346	4.95E−13
	Putative heme d1 biosynthesis protein NirF	−1.432629258	1.70E−11
	Cytochrome c-551	−1.357630256	7.11E−11
	Cytochrome c family protein	−1.197219265	2.16E−10
	Heme d1 biosynthesis radical SAM protein NirJ	−1.463653527	3.50E−10
	Nitric oxide reductase subunit B	−1.229229957	6.42E−09
	Nitric oxide reductase cytochrome c subunit NorC	−1.257119358	1.34E−08
	Nitrite reductase	−1.097383375	2.67E−07
	HemY	−0.959588379	1.43E−06
	Ubiquinone biosynthesis accessory factor UbiT	−1.155158561	1.69E−06
	Cytochrome-c oxidase%2C cbb3-type subunit III	−1.054265288	2.49E−06
	Cytochrome c family protein	−1.014018612	2.78E−06
	Cytochrome c oxidase assembly protein	−0.89668222	2.70E−05
	Pyruvate kinase	−1.919820581	0.004318579
	Cytochrome c domain-containing protein	−1.059927273	0.0470698

Down-regulated DEGs were grouped into functional categories based on Gene Ontology annotations, and the most represented biological categories were identified. Within each category, genes showing the strongest down-regulation are reported together with their log_2_FC and adjusted *p*-values.

Genes linked to aerobic and anaerobic respiration included those encoding enzymes of glycolysis and the Krebs cycle, components of the electron transport chain (ETC), their associated maturation factors, soluble transporters, and enzymes involved in heme biosynthesis. The main down-regulated components of the ETC were cytochromes involved in both aerobic respiration and denitrification. The most pronounced down-regulation within this functional category was observed for the 4Fe–4S ferredoxin-type domain-containing protein and cytochrome c oxidase.

#### 3.2.3 Metabolic and functional reprogramming of PF05 during fungal interaction

**Figure 3:**
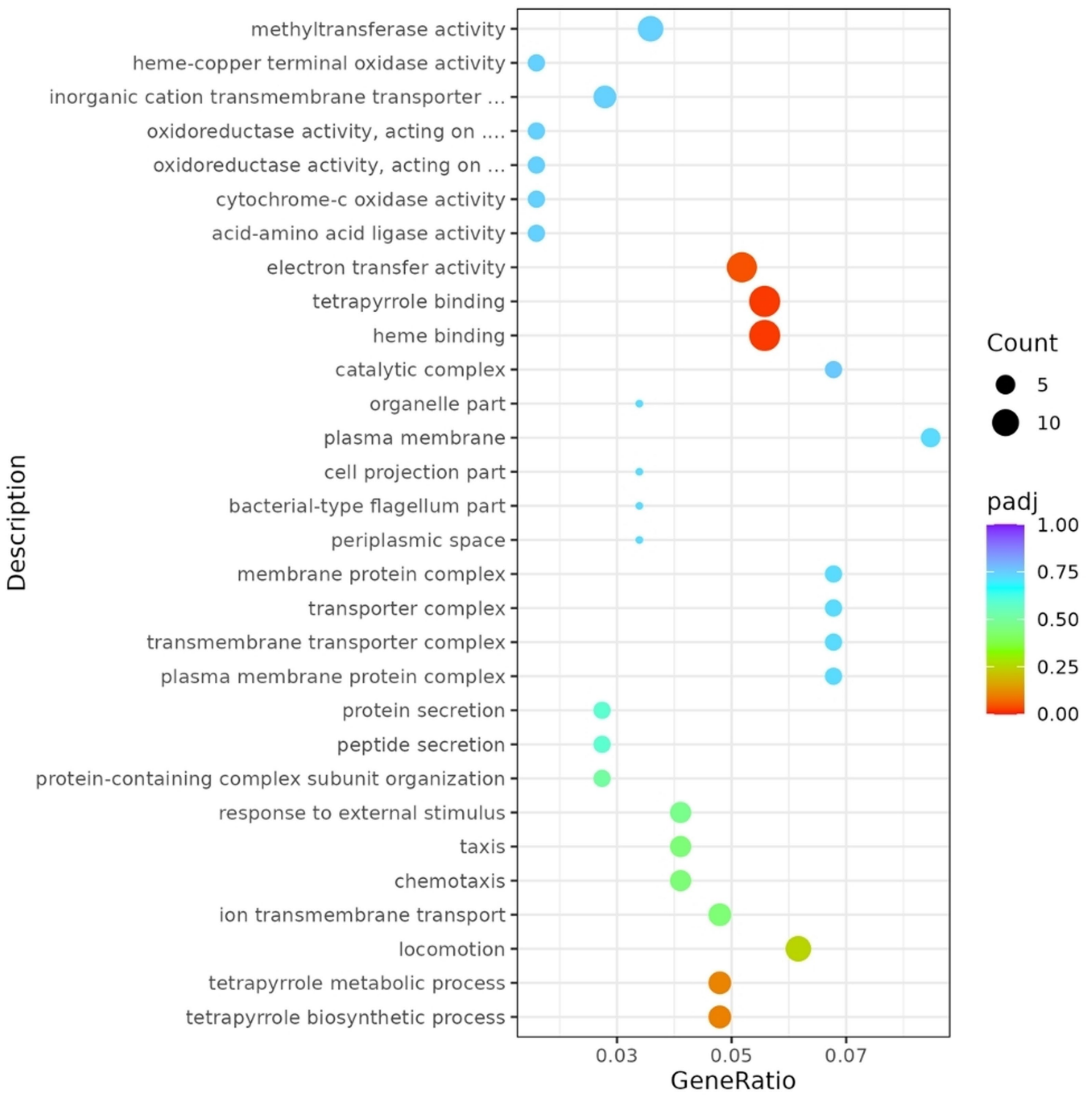
Dot plots of the top 30 enriched GO terms for down-regulated DEGs in Pseudomonas sp. PF05_DP vs PF05, grouped into the three main GO categories: Biological Process (BP), Cellular Component (CC), and Molecular Function (MF). The *x*-axis represents the GeneRatio, calculated as the proportion of DEGs annotated to a given GO term relative to the total DEGs within that category. The *y*-axis lists the top 30 GO terms. Dot size reflects the number of DEGs associated with each term, while dot color denotes the adjusted *p*-value (padj), ranging from red (more significant) to blue (less significant).

Gene Ontology (GO) enrichment analysis highlighted distinct functional trends between up- and down-regulated DEGs. For up-regulated genes, the most enriched GO terms were related to transmembrane transport, oxidoreductase activity, and small-molecule metabolic processes, although none reported statistical significance after multiple testing correction (data not shown). In contrast, down-regulated DEGs showed significant enrichment for electron transfer activity, heme binding, and tetrapyrrole binding, consistent with repression of respiratory and redox-associated functions (Figure 3).

**Figure 4:**
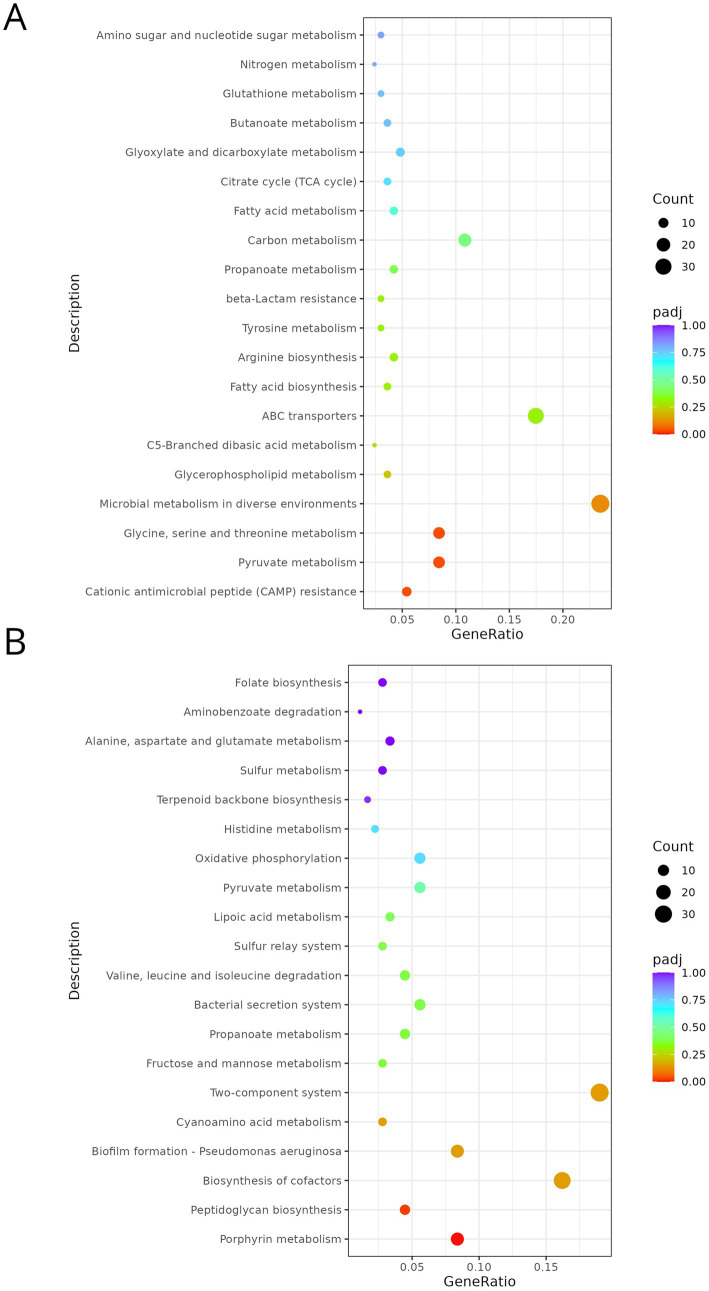
Dot plots of the top 20 enriched KEGG pathways associated with up- **(A)** and down-regulated **(B)** genes in *Pseudomonas fluorescens* PF05_DP vs. PF05. The *x*-axis represents the GeneRatio, calculated as the proportion of DEGs annotated to a given pathway relative to the total number of genes successfully mapped to KEGG within each category. The *y*-axis lists the top 20 enriched pathways. Dot size reflects the number of DEGs associated with each pathway, while dot color denotes the adjusted *p*-value (*padj*), ranging from red (more significant) to blue (less significant).

KEGG pathway enrichment analysis further supported these trends (Figure 4). Among up-regulated DEGs, CAMP resistance, pyruvate metabolism, and glycine, serine and threonine metabolism were the only significantly enriched pathways. Additional pathways related to microbial metabolism, carbon metabolism, and ABC transporters were also represented, although below the significance threshold. Down-regulated DEGs showed significant enrichment for porphyrin metabolism and peptidoglycan biosynthesis, with moderate enrichment of pathways linked to two-component systems, cofactor biosynthesis, and biofilm formation.

#### 3.2.4 Gene set enrichment analysis highlights coordinated regulation of iron homeostasis and antimicrobial defense

**Figure 5:**
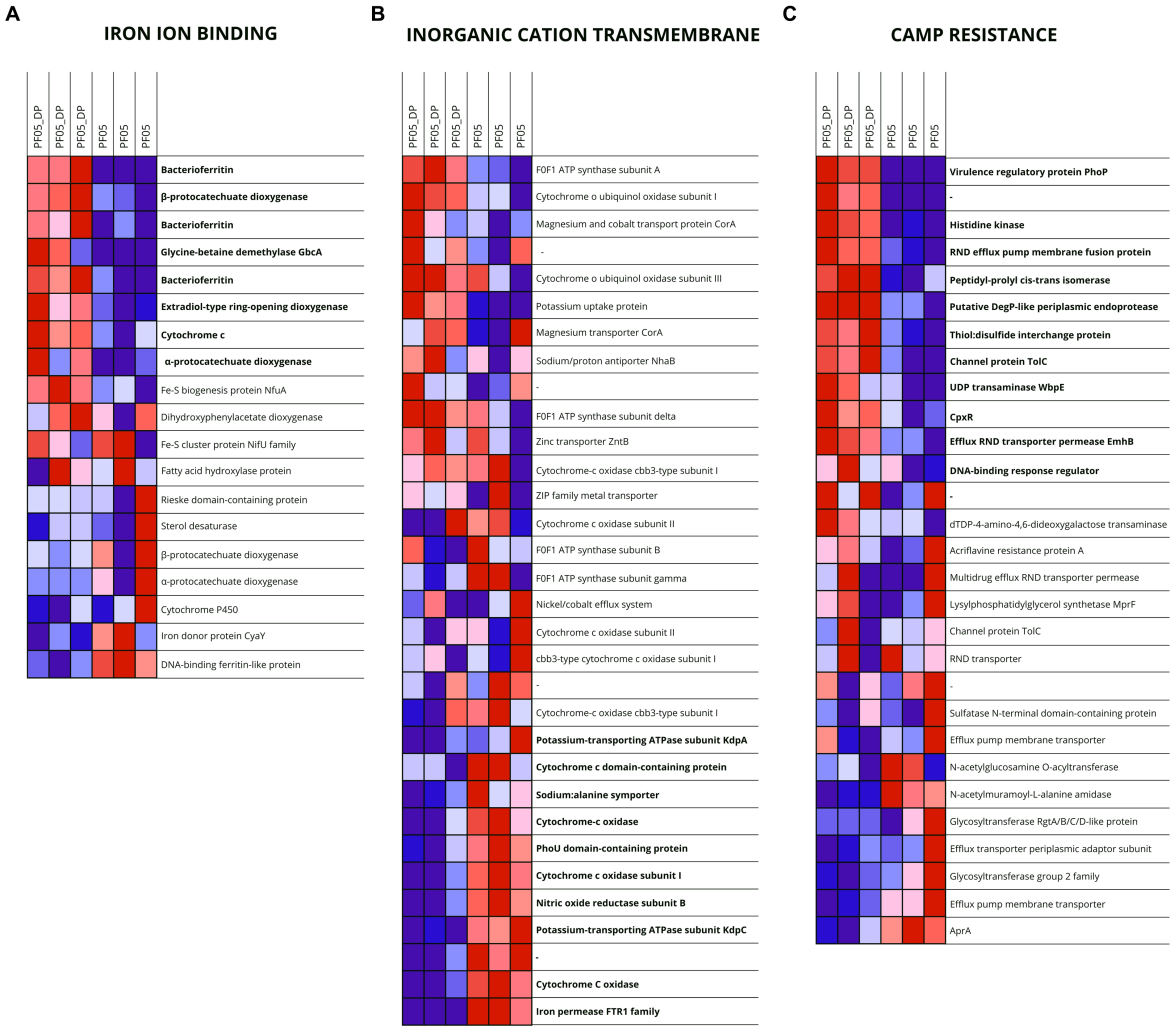
Expression profiles of genes associated with selected enriched functional gene sets, including iron ion binding, inorganic cation transmembrane transporter activity and cationic antimicrobial peptide resistance, in *Pseudomonas* sp. PF05_DP compared with PF05. Panels **(A–C)** correspond to the three functional gene sets listed above. Heatmaps display expression levels across biological replicates for each condition. Warmer colors indicate higher expression, whereas cooler colors indicate lower expression.

Gene Set Enrichment Analysis (GSEA) identified coordinated transcriptional responses not captured by single-gene enrichment approaches. Several gene sets were significantly positively enriched in PF05_DP, including those related to efflux transmembrane transporter activity, cellular catabolic processes, organonitrogen compound catabolism, and iron ion binding. Notably, this latter gene set (Supplementary Figure S1) showed strong positive enrichment, driven by oxidoreductases and iron-storage proteins, such as dioxygenases and bacterioferritins, indicating enhanced iron-associated metabolic activity during fungal interaction (Figure 5).

Conversely, a single gene set was significantly negatively enriched in PF05_DP, corresponding to inorganic cation transmembrane transporter activity (Supplementary Figure S2). Core genes within this set included proteins involved in respiration and transport of iron, sodium and potassium, suggesting coordinated repression of inorganic cation transport and respiratory activity (Figure 5).

GSEA performed on KEGG pathways identified several positively enriched gene sets, notably CAMP resistance, pyruvate metabolism, and tyrosine metabolism. The CAMP resistance gene set (Supplementary Figure S3) was dominated by regulators and efflux-associated proteins, including PhoP, CpxR, OmpR-family regulators, and outer membrane and membrane fusion proteins, all showing increased expression in PF05_DP. A single KEGG pathway, cyanoamino acid metabolism, was negatively enriched, driven by repression of genes involved in cyanide biosynthesis (*hcnABC*) (Figure 5).

#### 3.2.5 Comparative transcriptomic differences between PF4.89 and PF05 under basal conditions

Supplementary Table S4 reports the full list of shared genes found to be equally expressed, up- or down-regulated in *P. frederiksbergensis* PF4.89 and *Pseudomonas* sp. PF05, ordered by increasing *p*-value and adjusted *p*-value. Differential expression analysis comparing *P. frederiksbergensis* PF4.89 and *Pseudomonas* sp. PF05 grown in the absence of *Fusarium oxysporum* identified 2,534 differentially expressed genes (DEGs) (|log_2_FC| ≥ 1, adjusted *p* < 0.05), including 1,249 up-regulated and 1,285 down-regulated genes in PF4.89, while 1,171 genes showed no significant change in expression. Functional annotation based on Gene Ontology revealed extensive transcriptomic remodeling in PF4.89 relative to PF05, affecting multiple cellular processes.

Among genes up-regulated in PF4.89, those encoding ABC transporters and permeases were particularly abundant and clearly outnumbered their down-regulated counterparts. Genes involved in protein translation, including multiple ribosomal components, were also over-represented among up-regulated DEGs, suggesting enhanced expression of translational machinery. In addition, PF4.89 showed increased expression of genes linked to iron storage and stress tolerance, including bacterioferritins, as well as a member of the fusaric acid resistance protein family, although the latter displayed a modest log_2_ fold change (log_2_FC = 0.98). Notably, the outer membrane protein H1 was among the most strongly induced genes across the entire dataset (log_2_FC = 5.33).

Several functional categories exhibited a more balanced distribution between up- and down-regulation. Genes encoding outer membrane proteins, multidrug efflux systems (including RND pumps), and enzymes involved in central metabolism—notably carbohydrate, nitrogen, and lipid metabolism—were detected in both expression directions. Similarly, antioxidant systems displayed a heterogeneous pattern, with catalases and glutathione transferases predominantly up-regulated, whereas peroxiredoxins were exclusively down-regulated, indicating a complex reorganization of oxidative stress responses.

In contrast, other functional categories were predominantly down-regulated in PF4.89. These included members of the Major Facilitator Superfamily (MFS), genes associated with secretion systems, particularly the type VI secretion system, and numerous components of cellular respiration, including cytochromes and enzymes involved in heme biosynthesis. Genes implicated in iron acquisition and utilization, as well as those encoding peptidases and lipases, were likewise more frequently repressed.

From a regulatory standpoint, PF4.89 exhibited exclusive down-regulation of multiple LuxR-family transcriptional regulators and of the small RNAs Pseudomonas sRNA P31 and *Pseudomonas* sRNA P14, the latter representing the most strongly repressed transcript in the entire dataset (log_2_FC = −10.56).

Overall, the basal transcriptional profile of PF4.89 relative to PF05 was characterized by a broad but non-symmetric reprogramming, with selective activation of transport and translational functions accompanied by coordinated repression of respiration, secretion, regulatory, and iron-associated pathways.

## 4 Discussion

This study combined comparative genomics and transcriptomics to elucidate the molecular basis underlying the contrasting biocontrol performance of *Pseudomonas* sp. PF05 and *P. frederiksbergensis* PF4.89 against *F. oxysporum* (Di Rico et al., 2025). Genomic analyses revealed that PF05 harbors an expanded repertoire of genes associated with phenazine metabolism, polyketide biosynthesis, and non-ribosomal peptide-related functions compared with PF4.89. Although no single gene could be unequivocally identified as the sole determinant of antifungal activity, the combined presence of these pathways suggests a greater secondary metabolite biosynthetic potential that likely contributes to the biocontrol phenotype of PF05 as compared to PF4.89.

Interestingly, transcriptomic analysis revealed that during direct interaction with *F. oxysporum*, PF05 did not show a significant increase in the transcription of canonical antifungal secondary metabolite biosynthetic genes, including those involved in phenazine and non-ribosomal peptide production. Although several of these genes were expressed, their transcriptional levels did not differ significantly from the control condition. Importantly, these findings indicate that antifungal biosynthetic pathways remained transcriptionally active under the tested conditions, reflecting a preserved inhibitory potential capable of effectively controlling fungal growth. Consistent with this interpretation, previous studies have shown that microbial interaction does not necessarily alter the transcription of most antibiosis-related genes, while antimicrobial diversity and biocontrol capacity can be maintained independently of transcriptional variation (Luna-Bulbarela et al., 2024). At the same time, PF05 activated a broader adaptive response characterized by cellular protection, detoxification, and metabolic reprogramming, indicating a coordinated physiological adjustment to fungal-derived stress (Hennessy et al., 2017). A central component of this response was the strong up-regulation of transport and efflux systems. During interaction with *F. oxysporum*, PF05 showed increased expression of several genes encoding ABC transporters, permeases and members of the MFS, which are known to mediate the transport of a wide range of substrates. Under these conditions, their up-regulation suggests a role in the removal of toxic substances generated during fungal challenge (Diao et al., 2021; Hennessy et al., 2017). Among these membrane-associated defense mechanisms, the protein containing a fusaric acid resistance domain exhibited particularly pronounced expression levels, pointing to a key role in counteracting fusaric acid. This mycotoxin, produced by several *Fusarium* species, is known to suppress biocontrol activity in *Pseudomonas* by inhibiting the production of important secondary metabolites such as DAPG (Biessy and Filion, 2021; Quecine et al., 2015).

Adaptation to the chemically hostile environment imposed by *F. oxysporum* was also reflected in the strong overexpression of the outer membrane protein H1, previously linked to resistance to polymyxin B, aminoglycosides, and EDTA in *Pseudomonas protegens* (Bell and Hancock, 1989), together with a weak activation of RND-family efflux pumps (log_2_FC = 0.79), which likewise appeared to contribute to this protective response, in line with their established roles in antibiotic resistance, virulence, and solvent tolerance in Gram-negative bacteria (Adebusuyi and Foght, 2011).

In addition to transport-related defense, PF05 underwent a marked metabolic reprogramming to support stress tolerance and detoxification processes. Several oxidoreductases were up-regulated, indicating a coordinated detoxification response operating at multiple levels. In particular, the induction of quinone oxidoreductases, methionine sulfoxide reductases, and glutathione-dependent enzymes points to an enhanced capacity to counteract oxidative stress and limit cellular damage caused by redox-active fungal metabolites. These enzymes may also mitigate oxidative stress generated endogenously during antagonistic activity, thereby protecting PF05 from self-inflicted redox damage.

In parallel, the up-regulation of oxidoreductases involved in the degradation of aromatic and phenolic compounds supports the idea that PF05 also directly catabolizes fungal secondary metabolites, thereby reducing their toxicity and weakening fungal competitiveness. Notably, among these enzymes was quercetin 2,3-dioxygenase, known to act on a wide range of flavonoids, suggesting that PF05 may actively target phenolic compounds released during fungal interaction (Hennessy et al., 2017). This interpretation is consistent with the well-documented capacity of *F. oxysporum* to produce a wide range of secondary metabolites, including aromatic and redox-active compounds such as quinones, xanthones, anthranilates, and alkaloids, many of which display phytotoxic and antimicrobial activities (Ibrahim et al., 2021).

This detoxification-oriented response was further reinforced by the induction of enzymes such as (hydroxymethyl)benzenesulfonate dehydrogenase, involved in the degradation of aromatic xenobiotics, and 4-hydroxybenzoate 3-monooxygenase, a flavoprotein catalyzing the hydroxylation of phenolic compounds (Chenprakhon et al., 2019). Also noteworthy was a carboxymuconolactone decarboxylase-like protein of the β-ketoadipate pathway, which has been associated with bacterial resistance to several antibiotics with aromatic structures (Rana et al., 2023).

Metabolic reprogramming in PF05 also extended to acetoin and 2,3-butanediol metabolism. These volatile compounds are known to exert antifungal activity and to trigger induced systemic resistance in plants, suggesting that PF05 may simultaneously antagonize the pathogen and prime host defenses. The coordinated induction of both biosynthetic and degradative pathways for these metabolites further indicates tight metabolic regulation rather than indiscriminate overproduction (Ali and Moon, 2025).

Among genes associated with small-molecule metabolism, fumarylacetoacetase was of particular interest, as it has recently been associated with the Pseudomonas quinolone signal (PQS) quorum-sensing system, which regulates the production of key biocontrol-related secondary metabolites such as phenazines and hydrogen cyanide (Dutta et al., 2020).

Regulatory networks played a central role in shaping this adaptive response. LuxR-family regulators and the PhoPQ two-component system emerged as key elements controlling stress responses, transport, and quorum sensing. Enrichment of gene sets associated with resistance to cationic antimicrobial peptides further underscores the activation of membrane remodeling and defense mechanisms, reinforcing the notion that PF05 responds to fungal challenge through integrated regulatory control rather than isolated pathway induction.

Conversely, PF05_DP displayed a coordinated repression of genes involved in respiration, iron uptake, energy-intensive transport and secretion systems. Down-regulation of iron permeases, heme biosynthesis enzymes and cytochromes, together with the up-regulation of bacterioferritins, suggests a strategic limitation of intracellular iron availability to prevent oxidative damage. Reduced respiratory activity likely lowered ROS generation, allowing PF05 to operate under oxidative stress conditions imposed by the fungal interaction (Larosa and Remacle, 2018). This energy-saving strategy, coupled with selective transporter expression, indicates a fine balance between metabolic efficiency and stress resistance.

The comparative transcriptomic analysis under basal conditions further highlighted fundamental differences between PF05 and PF4.89. PF4.89 exhibited a transcriptional profile oriented toward nutrient uptake and cellular homeostasis, with enhanced expression of ABC transporters and translational machinery but reduced activation of secretion systems, regulatory networks, and respiratory pathways. The down-regulation of LuxR-family regulators and small regulatory RNAs suggests limited engagement of complex adaptive circuits, which may constrain the strain’s ability to rapidly respond to biotic stress. Together, these features depict PF4.89 as a strain optimized for resource exploitation rather than antagonistic interaction, consistent with its reduced biocontrol performance.

Overall, our results indicate that effective biocontrol by *Pseudomonas* sp. PF05 relies on a combination of genomic potential and dynamic transcriptional reprogramming. Rather than constitutively overproducing antifungal metabolites, PF05 deploys an integrated strategy encompassing detoxification, metabolic flexibility, regulatory coordination, and selective energy conservation. This multifaceted response likely confers robustness and resilience during interaction with *F. oxysporum*, highlighting the importance of regulatory and metabolic traits alongside classical secondary metabolite pathways. These insights provide a refined framework for understanding microbial biocontrol mechanisms and may inform the selection and optimization of strains for sustainable plant disease management.

## 5 Conclusion

This study demonstrates that the biocontrol efficacy of *Pseudomonas* sp. PF05 against *Fusarium oxysporum* is supported by a combination of genomic features and adaptive transcriptional responses rather than by the induction of single antifungal pathways. Comparative genomic analysis showed that PF05 possesses a broader secondary metabolite-related gene repertoire than the closely related strain *P. frederiksbergensis* PF4.89, providing a genetic foundation for biocontrol potential. However, transcriptomic data revealed that during fungal interaction PF05 primarily relies on a coordinated stress-adaptive strategy involving detoxification, efflux, metabolic reprogramming, and regulatory control, rather than on the overexpression of canonical antifungal biosynthetic genes. The repression of respiration- and iron-dependent processes, coupled with the activation of iron storage, redox homeostasis, and membrane defense systems, suggests that PF05 adopts an energy-efficient and protective physiological state that enhances resilience in chemically hostile environments. These insights underscore the importance of combining genome-resolved and transcriptome-based approaches for the rational selection and optimization of MBCAs and provide a framework for identifying traits associated with robust and predictable biocontrol performance in agricultural systems.

## Data Availability

The original contributions presented in the study are publicly available. This data can be found here: NCBI BioProject, accession PRJNA1441524.

## Footnotes

1
http://www.broadinstitute.org/gsea/index.jsp
