# Supplementary material for: Integrative genomic and transcriptomic insights into the biocontrol activity of Pseudomonas sp. PF05 against Fusarium oxysporum
Source: Front Microbiol. 2026 Apr 10;17:1784504. doi: 10.3389/fmicb.2026.1784504 (PMC13106123; doi:10.3389/fmicb.2026.1784504)
Supplement: Supplementary file 5 [file Table_5.DOCX]

Supplementary Material

# SUPPLEMENTARY TABLES AND FIGURES

## Figures


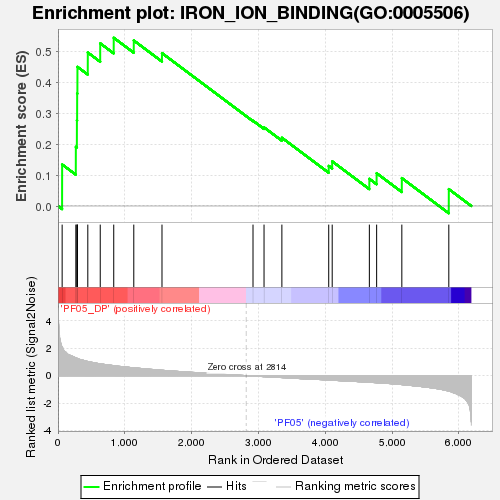


**Figure S1.** Gene set enrichment analysis (GSEA) of the GO term *iron ion binding* in the comparison between *Pseudomonas* sp. PF05_DP and PF05. The enrichment plot shows the running enrichment score (ES) (green line), the positions of genes belonging to the gene set along the ranked list (black vertical lines), and the color bar reflecting correlation with PF05_DP (red) or PF05 (blue).


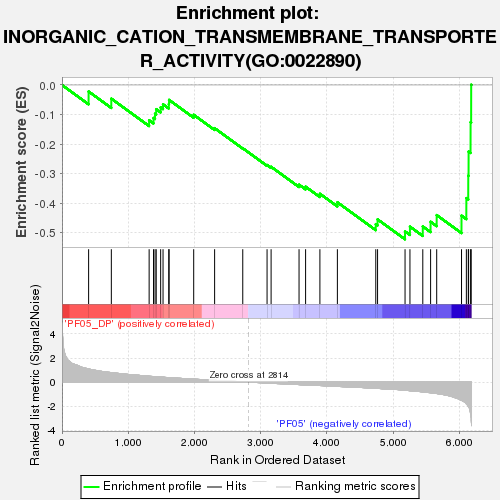


**Figure S2.** Gene set enrichment analysis (GSEA) of the GO term *inorganic cation transmembrane transporter activity* in the comparison between *Pseudomonas* sp. PF05_DP and PF05. The enrichment plot shows the running enrichment score (ES) (green line), the positions of genes belonging to the gene set along the ranked list (black vertical lines), and the color bar reflecting correlation with PF05_DP (red) or PF05 (blue).


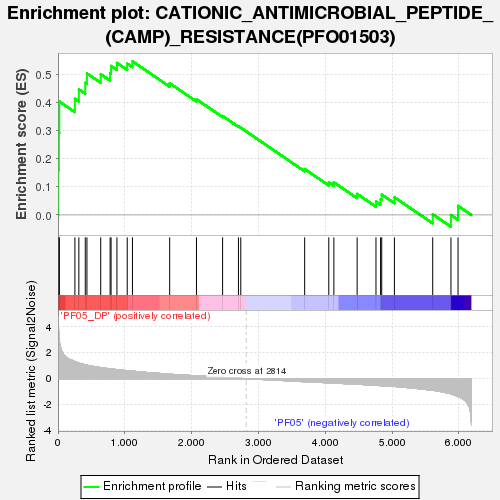


**Figure S3.** Gene set enrichment analysis (GSEA) of the GO term *cationic antimicrobial peptide (CAMP) resistance* in the comparison between *Pseudomonas* sp. PF05_DP and PF05. The enrichment plot shows the running enrichment score (ES) (green line), the positions of genes belonging to the gene set along the ranked list (black vertical lines), and the color bar reflecting correlation with PF05_DP (red) or PF05 (blue).

## Table

**Table S1.** Hierarchical classification of plant growth-promoting traits (PGPTs), including putative PGPT-associated genes, identified in *Pseudomonas* sp. PF05 using the PLaBAse pipeline in strict mode. The table reports the full functional annotation structure from level 1 to level 6, together with the frequency of associated genes (freq) and their relative percentage (%) within the total PGPT hits. PGPTs include both direct and indirect plant growth-promoting functions, encompassing bio-fertilization, plant colonization, competitive exclusion, stress control and biocontrol-related traits, phytohormone and plant signal production, bio-remediation, as well as stimulation of plant immune responses.

**Table S2.** Hierarchical classification of plant growth-promoting traits (PGPTs), including putative PGPT-associated genes, identified in *Pseudomonas frederiksbergensis* PF4.89 using the PLaBAse pipeline in strict mode. The table reports the full functional annotation structure from level 1 to level 6, together with the frequency of associated genes (freq) and their relative percentage (%) within the total PGPT hits. PGPTs include both direct and indirect plant growth-promoting functions, encompassing bio-fertilization, plant colonization, competitive exclusion, stress control and biocontrol-related traits, phytohormone and plant signal production, bio-remediation, as well as plant immune response stimulation-related traits.

**Table S3.** Full list of all equally expressed and differentially expressed genes (up- and down-DEGs) in *Pseudomonas* sp. PF05_DP compared with PF05. The table reports gene IDs and annotations, normalized expression values for each biological replicate, log2 fold change, *p*-values and adjusted *p*-values, together with genomic features and functional descriptions.

**Table S4.** Full list of all equally expressed and differentially expressed shared genes (up- and down-DEGs) in *P*. *frederiksbergensis* PF4.89 and *Pseudomonas* sp. PF05. The table reports gene IDs and annotations, normalized expression values for each biological replicate, log2 fold change, *p*-values and adjusted *p*-values, together with genomic features and functional descriptions.
